# A Novel Splice-Site Variant in *CACNA1F* Causes a Phenotype Synonymous with Åland Island Eye Disease and Incomplete Congenital Stationary Night Blindness

**DOI:** 10.3390/genes12020171

**Published:** 2021-01-27

**Authors:** Usman Mahmood, Cécile Méjécase, Syed M. A. Ali, Mariya Moosajee, Igor Kozak

**Affiliations:** 1 Department of Ophthalmology, Hull Royal Infirmary, Hull 62807, UK; visionusman@gmail.com; 2 UCL Institute of Ophthalmology, London EC1V 9EL, UK; c.mejecase@ucl.ac.uk (C.M.); m.moosajee@ucl.ac.uk (M.M.); 3 Moorfields Eye Hospital UAE, Abu Dhabi 62807, United Arab Emirates; syed.ali@moorfields.ae; 4 Moorfields Eye Hospital NHS Foundation Trust, London EC1V 2PD, UK; 5 Great Ormond Street Hospital for Children NHS Foundation Trust, London WC1N 3JH, UK; 6 The Francis Crick Institute, London NW1 1AT, UK

**Keywords:** *CACNA1F* retinopathy, Åland island eye disease, congenital stationary night blindness

## Abstract

Background: *CACNA1F*-related disorders encompass progressive and non-progressive disorders, including Åland island eye disease and incomplete congenital stationary night blindness. These two X-linked disorders are characterized by nystagmus, color vision defect, myopia, and electroretinography (ERG) abnormalities. Ocular hypopigmentation and iris transillumination are reported only in patients with Åland island eye disease. Around 260 variants were reported to be associated with these two non-progressive disorders, with 19 specific to Åland island eye disease and 14 associated with both Åland island eye disease and incomplete congenital stationary night blindness. *CACNA1F* variants spread on the gene and further analysis are needed to reveal phenotype-genotype correlation. Case Report: A complete ocular exam and genetic testing were performed on a 13-year-old boy. A novel splice-site variant, c.4294-11C>G in intron 36 in *CACNA1F*, was identified at hemizygous state in the patient and at heterozygous state in his asymptomatic mother and explained the phenotype synonymous with Åland island eye disease and incomplete congenital stationary night blindness observed in the patient. Conclusion: We present a novel variant in the *CACNA1F* gene causing phenotypic and electrophysiologic findings indistinguishable from those of AIED/CSNB2A disease. This finding further expands the mutational spectrum and our knowledge of *CACNA1F*-related disease.

## 1. Introduction

Åland island eye disease (AIED; OMIM #300600), also known as Forsius-Eriksson type ocular albinism, is an X-linked disorder first described in Norwegian descendants on the Åland Islands in the Sea of Bothnia [1]. It is a rare disease of unknown incidence. Affected males have nystagmus, myopia, reduced visual acuity, red-green color vision deficits, iris trans-illumination defects, foveal hypoplasia, and a blonde fundus without evidence of chiasmal misrouting [1,2]. Electroretinography (ERG) reveals defects in a reduced photopic function and scotopic b-wave amplitude (Schubert-Bornschein type) [3]. However, AIED is considered stationary, except for progressive axial myopia [4,5,6]. 

AIED was initially thought to be a variant of X-linked ocular albinism, type 1, Nettleship-Falls type (OMIM #300500) [1]; however, latent nystagmus of extraocular origin, absence of macromelanosomes in skin biopsy specimens, and no evidence of intracranial chiasmal misrouting differentiates the conditions [2,7]. Female carriers do not show any features of the disease, except for slight latent nystagmus in some cases [6].

The AIED gene locus has been localized to the pericentromeric region of the Xp11.23 chromosome, Xp11.23; shared with incomplete congenital stationary night blindness (CSNB2A, OMIM #300071) [8]. These diseases share numerous clinical features. X-linked CSNB2A is a non-progressive condition characterized by nyctalopia, myopia, nystagmus, strabismus, and a negative ERG. Both AIED and CSNB2A result from variants in the calcium channel, voltage-dependent, α1F-subunit gene, *CACNA1F* (MIM 300110) [9], and have been suggested to be indistinct with genetic or environmental modifiers influencing the phenotypic variation. A novel missense variant (c.1807G>C, p. (Gly603Arg)) in *CACNA1F* has been reported in one family to cause both AIED and CSNB2A within affected members, providing further evidence for this [10]. Moreover, comparison of the clinical features of both diseases suggests that AIED-like disease and CSNB2A may be identical allelic disorders [11]. The following clinical features overlap between AIED and CSNB2A: decreased visual acuity, nystagmus, astigmatism, defective dark adaptation, and ERG abnormalities in both photopic and scotopic functions. The distinguishing clinical features between the two include fundus hypopigmentation, progressive myopia, and protan color vision defect [12]. 

*CACNA1F* (calcium channel, voltage-dependent, alpha-1F subunit) is located on chromosome Xp11.23 and consists of 48 exons spanning a genomic region of 28 kb. This gene encodes a multipass transmembrane protein of 1977 amino acids that functions as an alpha-1 subunit of the voltage-dependent calcium channel, which mediates the influx of calcium ions into the cell. Expressed throughout the retina in the outer nuclear, inner nuclear, and ganglion cell layers of the retina [9,13], these channels support Ca^2+^ influx under relatively depolarized conditions, which is necessary for tonic glutamate release from rod and cone photoreceptors [10]. CACNA1F protein contains four homologous domains (I–IV); each domain comprises six transmembrane helical segments (S1–S6) and forms the pore that permits ions to flow down the electrochemical gradient from the extracellular milieu into the cytoplasm [14].

Herein, we present a clinical case with novel hemizygous splice variant (c.4294-11C>G) in the *CACNA1F* gene at an evolutionarily highly conserved position that is predicted to be pathogenic by all applied in silico algorithms. 

## 2. Case Description

**Figure 1:**
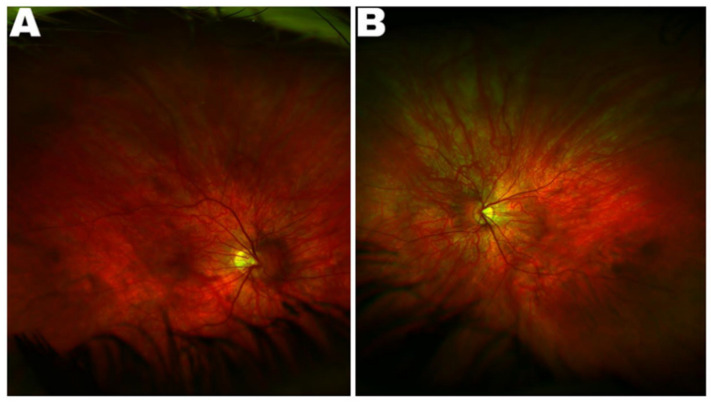
Ultrawide-field color fundus photograph of proband’s right eye (**A**) and left eye (**B**) showing hypopigmented fundus with peripapillary atrophy.

A 13-year-old Caucasian boy presented with reduced vision. There was no family history of consanguinity or nystagmus in family members. His past medical history was unremarkable and he has had slowly progressive myopia in both eyes. His best corrected visual acuity (BCVA) was 0.5 (logMAR) in each eye. He had high myopia with a current refraction—7.5SD/+1.5SC at 60 degrees in both eyes. Clinical examination revealed horizontal jerk nystagmus with no significant head posture. Anterior segment examination was normal with no signs of iris transillumination. Fundus examination showed hypopigmented (blonde) fundi with visible choroidal vasculature and peripapillary atrophy with a mildly hypoplastic optic nerve in both eyes (Figure 1) which was confirmed with magnetic resonance imaging (MRI) of the patient’s brain and orbits.

**Figure 2:**
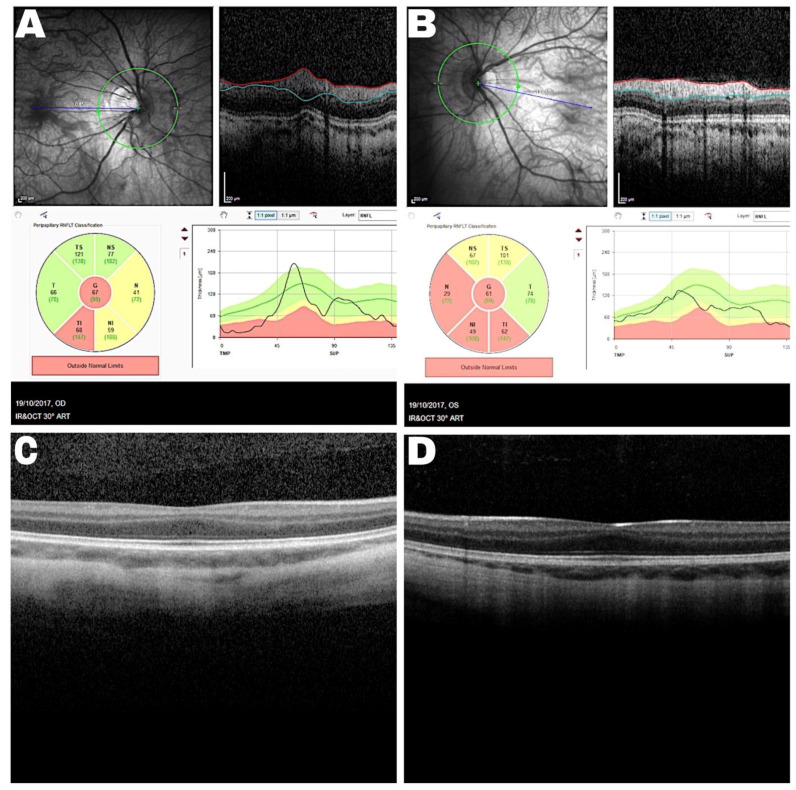
Spectral domain optic coherence tomography (SD-OCT) of the proband showing nerve fiber layer thinning in the right (**A**) and left (**B**) eye. SD-OCT macular scan demonstrates fovea plana in the right (**C**) and left eye (**D**).

Spectral domain optical coherence tomography (SD-OCT) showed bilateral foveal hypoplasia, grade 1, displaying presence of the inner plexiform and inner nuclear layers at the fovea with a widened outer nuclear layer (Figure 2). In addition, the retinal nerve fiber layer showed some thinning, but Goldmann perimetry showed no constriction of either visual field. Fundus autofluorescence showed a reduced foveal reflex but was otherwise normal.

Full-field electroretinography (ERG) examination was done using skin electrodes as the patient did not tolerate corneal ones. Photopic flash and flicker responses (Light Adapted 3.0) were severely attenuated in both eyes. Isolated rod responses (Dark Adapted (DA) 0.01) were also severely attenuated bilaterally. Combined rod cone (DA 3.0) and responses to high flash (DA 10.0) demonstrated normal a-waves (for pad recording); however, b-wave was markedly attenuated giving rise to an electronegative waveform in both eyes, characteristically known as Schubert-Bornschein type ERG. The multichannel pattern onset visual evoked potentials (VEP) demonstrated symmetrical responses and normal responses across right and left occipital recording when each eye was tested independently.

**Figure 3:**
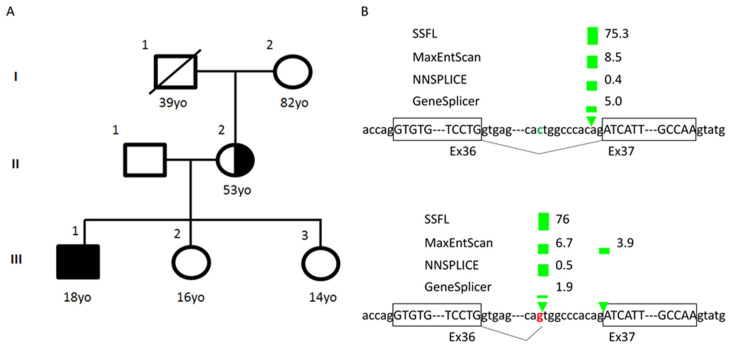
(**A**) Family pedigree showing proband and mother as a female carrier. (**B**) Several websites (SpliceSite Finder-like, MaxEntScan, NNSPLICE, and GeneSplicer, via Alamut software) show reference sequence (upper panel) and predict the loss of normal splice-site acceptor due to c.4294-11C>G variant (lower panel).

The proband underwent a targeted gene panel for congenital stationary night blindness containing 16 genes (*CABP4*, *CACNA1F*, *CACNA2D4*, *GNAT1*, *GPR179*, *GRK1*, *GRM6*, *LRIT3*, *NYX*, *PDE6B*, *RBP4*, *RDH5*, *RHO*, *SAG*, *SLC24A1*, *TRPM1*) through the Oxford University Hospitals NHS Foundation Trust Genetic Laboratory at Oxford, UK. Mutation screening was carried out by next generation sequencing with library preparation using the Agilent focused clinical exome +1 kit followed by sequencing on the Illumina platforms. Data were analyzed using an in-house pipeline and virtual gene panels, with all mutations confirmed by Sanger sequencing. The proband was found to have a novel hemizygous splice-site mutation c.4294-11C>G in intron 36 of the *CACNA1F* gene (hg19; chrX:g.49067563G>C, NM_005183.2:c.4294-11C>G) (Figure 3). This variant has been predicted to affect the efficiency of the splice acceptor site and lead to aberrant splicing. Parental segregation found that the mother was a carrier of the variant; however, she was clinically asymptomatic with no phenotypic features of *CACNA1F*-related disease. Figure 3 depicts the family pedigree of the proband.

## 3. Discussion

**Figure 4:**
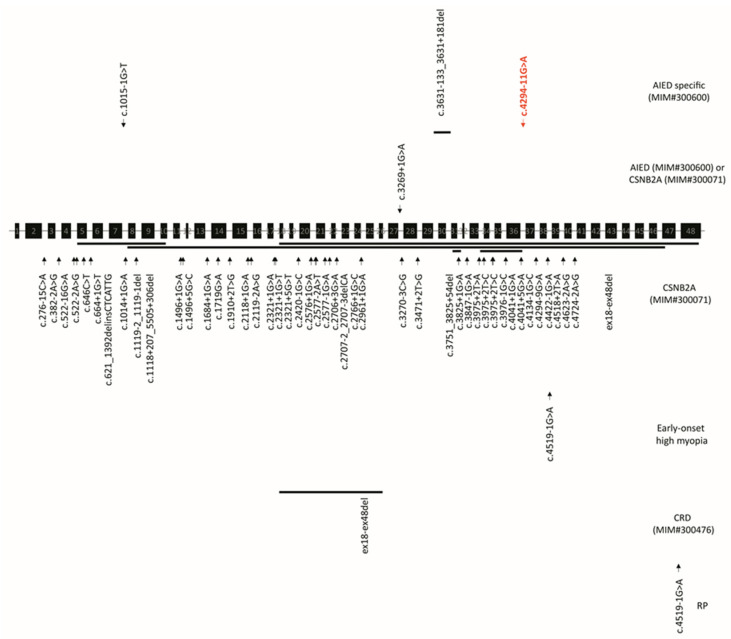
Disease-causing variants associated with *CACNA1F*-related disorders. Splice-site variants and large deletions/insertions are depicted in the 48 exons of *CACNA1F* (NM_005183.4). Red variant is reported in this study. Abbreviations: AIED—Åland island eye disease, CSNB2A—congenital stationary night blindness, CRD—cone-rod dystrophy, RP—retinitis pigmentosa.

**Figure 5:**
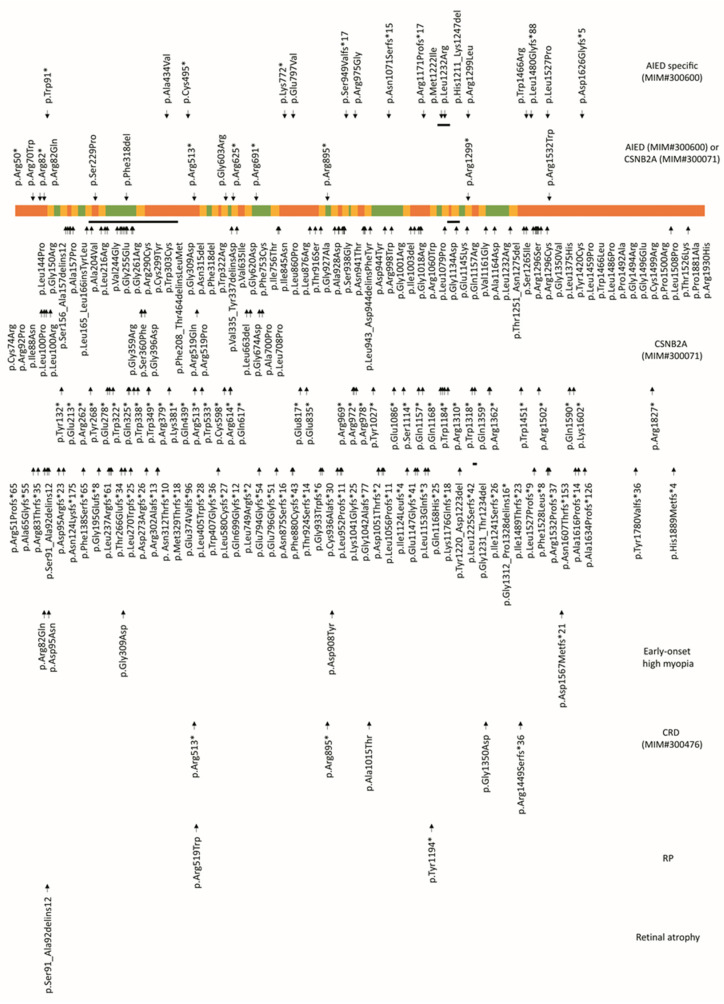
CACNA1F disease-causing variants are associated with ocular disorders. CACNA1F protein is composed of several cytoplasmic (orange), helical (yellow), and extracellular (green) regions (NP_005174.2; Uniprot O60840). Abbreviations: AIED—Åland island eye disease, CSNB2A—congenital stationary night blindness, CRD—cone-rod dystrophy, RP—retinitis pigmentosa.

*CACNA1F* variants have been reported to be associated with non-progressive diseases such as AIED and CSNB2A, and with progressive diseases like early-onset high myopia, retinitis pigmentosa, and cone/cone-rod dystrophy. Two-hundred and thirty variants were reported to be associated with CSNB2A, 19 were specific to AIED, and 14 were associated with both AIED and CSNB2A (Figure 4 and Figure 5).

There was no clear genotype-phenotype correlation. The variant identified in this study is located in intron 36, two nucleotides away from the splice-site variant c.4294-9G>A associated with CSNB2A. Interestingly, the splice variant c.4294-9G>A was reported to be associated with CSNB2A and leads to a misplicing in an in vitro model: a part of intron 36 is retained [15]. Compared with this CSNB2A variant, the c.4294-11C>G variant may lead to intron retention until the c.4295-10 position (Figure 3B). However, further analysis would be needed to confirm this aberrant splicing.

Mutations in this specific gene have been implicated in both X-linked AIED and CSNB2. Although AIED and CSNB2A have many overlapping clinical features, the former has been reported to have additional features such as progressive myopia, dyschromatopsia, iris trans-illumination defects, hypopigmented fundus, and foveal hypoplasia [16]. The unique phenotypic feature in the index case is hypopigmented fundus which initially suggested diagnosis of ocular albinism. SD-OCT demonstrated a shallow foveal pit, persistence of all the inner retinal layers, and widening of the outer nuclear layer described previously in both AIED and CSNB2A [17]. Per some reports, there are a few distinct differences between the symptoms of the patients in the original AIED family and those described as having CSNB2A, including optic nerve and retinal damage in the latter [18,19].

The ERG recordings in the index case were consistent with Schubert-Bornschein type ERG, which is characterized by a normal a-wave with an extremely reduced b-wave in the maximal response of the ISCEV standard protocol. There was no electrophysiologic evidence of crossed asymmetry on the pattern onset VEP and responses were normal in both eyes. The most striking finding, however, was the truly electronegative ERG findings in both eyes. In incomplete congenital stationary night blindness (CSNB), full-field ERG recordings show a diminished, but not completely abolished, dark adaptation ERG (one of the features that distinguishes the ERG phenotype from that seen in complete CSNB). The dark adaptation strong flash (DA10) ERG has a markedly electronegative waveform with normal or near-normal a-wave [20]. This was also seen in our patient. The light adaptation ERG findings are more severely affected than those seen in complete CSNB, due to dysfunction in both the ON and OFF cone bipolar pathways. The conjunction of these ERG findings in a male with an X-linked family history is almost diagnostic of *CACNA1F*-associated retinopathy [20]. 

It is apparent that the clinical and electrophysiologic characteristics of AIED and incomplete X-linked CSNB are very similar. It has been suggested that CSNB2A and AIED are allelic variants but may not be mutation-specific [10]. The clinical features of these two diseases seem to be nearly identical considering that clinical variability is not uncommon among patients with CSNB2A, even among patients with the same *CACNA1F* variant [21]. The phenotypic differences may be attributable to differences in genetic background (i.e., other genes, possibly genetic modifiers). Similarly, in our case, while some features such as fundus hypopigmentation and progressive myopia may favor AIED, it is difficult to differentiate the two diseases clinically or genetically.

In summary, we present a case with a novel variant in the *CACNA1F* gene causing phenotypic and electrophysiologic finding indistinguishable from those of AIED/CSNB2A disease. This finding further expands the mutational spectrum and our knowledge of *CACNA1F*-related disease. Numerous studies, including this report, suggest AIED and CSNB2A are part of the same clinical disease spectrum. To better represent this cohort of patients, we should consider classifying them as *CACNA1F*-oculopathy rather than using eponymous names. Further work is required to identify genetic modifiers that lead to the less frequent variable features. 

## Data Availability

Not applicable.
